# Releasing synthetic data from the Avon Longitudinal Study of Parents and Children (ALSPAC): Guidelines and applied examples

**DOI:** 10.12688/wellcomeopenres.20530.2

**Published:** 2024-12-24

**Authors:** Daniel Major-Smith, Alex S. F. Kwong, Nicholas J. Timpson, Jon Heron, Kate Northstone

**Affiliations:** 1 Population Health Sciences, Bristol Medical School, University of Bristol, Bristol, England, BS8 2BN, UK; 2 Division of Psychiatry, The University of Edinburgh, Edinburgh, Scotland, EH10 5HF, UK; 3 MRC Integrative Epidemiology Unit, University of Bristol, Bristol, England, UK

**Keywords:** ALSPAC, Synthetic data, Reproducibility, Confidentiality, Open science, Methods education

## Abstract

The Avon Longitudinal Study of Parents and Children (ALSPAC) is a prospective birth cohort. Since its inception in the early 1990s, the study has collected over thirty years of data on approximately 15,000 mothers, their partners, and their offspring, resulting in over 100,000 phenotype variables to date. Maintaining data security and participant anonymity and confidentiality are key principles for the study, meaning that data access is restricted to *bona fide* researchers who must apply to use data, which is then shared on a project-by-project basis. Despite these legitimate reasons for restricting data access, this does run counter to emerging best scientific practices encouraging making data openly available to facilitate transparent and reproducible research. Given the rich nature of the resource, ALSPAC data are also a valuable educational tool, used for teaching a variety of methods, such as longitudinal modelling and approaches to modelling missing data. To support these efforts and to overcome the restrictions in place with the study’s data sharing policy, we discuss methods for generating and making openly available synthesised ALSPAC datasets; these synthesised datasets are modelled on the original ALSPAC data, thus maintaining variable distributions and relations among variables (including missing data) as closely as possible, while at the same time preserving participant anonymity and confidentiality. We discuss how ALSPAC data can be synthesised using the ‘synthpop’ package in the R statistical programming language (including an applied example), present a list of guidelines for researchers wishing to release such synthesised ALSPAC data to follow, and demonstrate how this approach can be used as an educational tool to illustrate longitudinal modelling methods.

## Introduction

Scientific best practice is moving towards enhanced openness, reproducibility and transparency, with data and analysis code increasingly being shared alongside scientific publications ( Bouter, 2023; Localio *et al.*, 2018; Munafò *et al.*, 2017; Smaldino *et al.*, 2019). Although data sharing is still not universal, there is a continued push from academics, journals, funders and governments towards this goal ( Abbasi, 2023; Federer *et al.*, 2018; Hardwicke *et al.*, 2018; House of Commons Science Innovation and Technology Committee, 2023; Malički *et al.*, 2021; Mathur & Fox, 2023; Minocher *et al.*, 2021; Shepherd *et al.*, 2017; Tedersoo *et al.*, 2021). While beneficial for science as a whole, these changes may be challenging for research from certain sources, such as large-scale population-based longitudinal studies, where data often cannot be made openly available. Reasons for this include preserving participant anonymity and ensuring that only legitimate researchers are able to access the resource ( Colditz, 2009; Hogue, 1991; Quintana, 2020; Samet, 2009; Shepherd *et al.*, 2017). This is the case for the focus of this paper, the Avon Longitudinal Study of Parents and Children (ALSPAC). ALSPAC is a longitudinal population-based birth cohort which enrolled approximately 15,000 pregnant women resident in the Bristol area of the UK who had expected dates of delivery between 1st April 1991 and 31st December 1992. These women, their partners, and their children – and more recently these children’s children – have been followed ever since ( Boyd *et al.*, 2013; Fraser *et al.*, 2013; Lawlor *et al.*, 2019; Major-Smith *et al.*, 2023; Northstone *et al.*, 2019; Northstone *et al.*, 2023). The ALSPAC resource is available to *bona fide* researchers, and it is not possible to release observed data alongside published work (as detailed in the ALSPAC Data Management plan).

Rather than releasing observed data, an alternative approach to data sharing is based on creating ‘synthetic’ datasets (for an introduction to synthetic data, see; ( Raghunathan, 2021)). These synthesised datasets are modelled on the original observed data, thus closely maintaining the marginal distributions of variables (e.g., mean, variation, cell counts, etc.), as well as the relationships between variables. However, as data are simulated and do not correspond to real-life individuals by design, they preserve participant anonymity (note that, because fully synthetic datasets do not contain personal information of real individuals, they are likely exempt from complying to the European Union’s General Data Protection Regulation [GDPR]; Beduschi, 2024). Although various approaches to synthetic dataset creation exist ( Raghunathan, 2021), the methods followed here are based on the ‘synthpop’ package available in the R programming language, which has been spear-headed by a longitudinal studies group at the University of Edinburgh ( Nowok *et al.*, 2016) (see also https://www.synthpop.org.uk/about-synthpop.html).

While synthetic data may not exactly preserve the attributes of the original observed data, due to random variability and the inability of models to perfectly recreate the original data, any analyses and conclusions ought to be similar ( Nowok *et al.*, 2016; Quintana, 2020). This can enable readers of the paper – either pre-publication, during the peer review process or post-publication – to explore the raw data, understand the analyses better, and replicate analyses themselves using these synthesised data ( Coughlin, 2017; Quintana, 2020; Shepherd *et al.*, 2017). This can help provide readers with assurance that the reported results are broadly correct, allow readers to test out the methods, and could also help with the self-correction of science by noticing potential errors in the analyses (such as treating a categorical variable as continuous ( Decety *et al.*, 2015; Shariff *et al.*, 2016), or recoding the control and intervention groups of a clinical trial incorrectly ( Goldacre *et al.*, 2019), to give two high-profile examples). In addition, synthesised datasets from longitudinal studies such as ALSPAC could be created as an educational tool to help others learn about new and/or complex methods ( Goldstein, 2018); for an example using synthesised data to explore longitudinal growth trajectory modelling, see ( Elhakeem *et al.*, 2022).

In this paper we: i) briefly describe the ‘synthpop’ package in more detail; ii) discuss recommendations for checking the synthesised data and ensuring that synthesised data are non-disclosive; iii) introduce guidelines to be adopted by researchers wishing to release synthesised ALSPAC data; iv) provide an example workflow for synthesising ALSPAC data (using an openly-available ALSPAC dataset); and v) present an example of how synthetic ALSPAC data can be used as an educational tool, focusing on longitudinal modelling methods.

## Creating synthetic datasets using ‘synthpop’

The ‘synthpop’ package works sequentially, with each variable synthesised conditional on previously-synthesised variables (other than the first variable, which is synthesised based on random sampling from the observed values). For instance, say our dataset had just three variables: age, sex and height. If age was synthesised first, this would be generated by randomly sampling from the observed distribution of age. If sex were synthesised next, it would be generated conditional on the previously-synthesised variable ‘age’, generating synthetic observations by randomly sampling from the range of predicted values from this model in the observed data. Finally, if height was synthesised last, it would be generated conditional on both previously-synthesised variables ‘age’ and ‘sex’, with synthetic values again generated by randomly sampling from the range of predicted values from this model in the observed data. The default algorithm for synthesising data is tree-based (using classification and regression trees; CART), but it is also possible to synthesise data using alternative tree-based (e.g., random forest) or parametric (e.g., linear, logistic) models. Note also that this method accounts for missing data, and maintains relations between missing data and other variables ( Nowok *et al.*, 2016). This process of synthetic data generation is closely related to the method of multiple imputation by chained equations for imputing missing data ( van Buuren, 2018); the difference being that, rather than only imputing missing values, these synthetic data methods generate wholly-synthetic datasets based on the observed data ( Raghunathan, 2021).

Most of the synthesising is automated by the command ‘syn’ within the ‘synthpop’ package, although it is possible to specify various options, such as the type of model to use, the order in which variables are synthesised, the choice of predictor variables, whether to apply a ‘smoothing’ parameter to continuous variables (to lower the risk of disclosive data for continuous data), and applying rules to maintain relations between variables (for instance, if synthesising variables such as ‘ever smoked’ and ‘amount smoked per day’, one could specify a rule that said ‘if never smoked, then code amount smoked per day as 0’). For more information on the ‘synthpop’ package and its functionality, see ( Nowok *et al.*, 2016).

## Recommendations when using ‘synthpop’: An example in population-based studies

Successful synthesis should meet two goals: i) preserving participant anonymity; and ii) maintaining relations between variables, the latter being vital for reproducibility ( Quintana, 2020). These two goals may trade-off somewhat against one another, as we discuss below.

The most important factor to consider when synthesising data is the potential disclosure risk. As synthetic datasets are wholly simulated, data ought to be non-disclosive as they are no longer based on individual records. However, it is possible that a unique combination of values could be synthesised corresponding to a unique individual in the observed data, thus remaining a potential disclosure risk. Although researchers using the synthesised data will not be able to know whether such a unique observation matches that of an actual participant, there is a remote possibility that unique individuals could be identified from the synthesised data. We therefore recommend that users undertake ‘statistical disclosure control’ checks on any synthetic datasets to remove any unique observations that occur in both the observed and synthetic datasets. This can be done easily within the ‘synthpop’ package using the commands ‘replicated.uniques’ (to tabulate these cases) and ‘sdc’ (to remove these cases). In our experience, the number of such unique replicates within synthesised ALSPAC data is likely to be quite low. For instance, in the dataset introduced below with 15 variables and 3,727 observations, when using the default CART synthesising method, only 4 observations (0.11% of the original sample) were unique replicates which had to be removed. However, this does depend on a range of factors, such as the number and type of variables; for instance, because there is less variation in possible responses, a dataset with many categorical variables may be more likely to result in a greater number of unique replicates, especially if some categories have low cell counts (an example is given in the associated script where approx. 10% of synthesised cases are unique replicates; see the ‘Data Availability’ section for more information).

If the number of unique replicates is found to be higher than one would like or anticipate, there are a number of available options (although what constitutes a ‘high number’ of unique replicates is a subjective matter and is up to the researcher to decide). For instance, synthesising data via parametric, rather than tree-based, methods may reduce the number of such cases. For synthesising continuous data, a ‘smoothing’ option can be applied which may provide an additional level of disclosure control by making the synthesised values slightly different from the observed values. Top and bottom coding of variables is also available, which may help reduce any potentially identifiable outliers. For more information on statistical disclosure control in ‘synthpop’, see ( Nowok *et al.*, 2016). However, in some circumstances it may not be possible to significantly reduce the number of unique replicates, and hence the sample size of the observed vs synthetic data may differ quite substantially; as long as the remaining synthetic dataset maintains the relations between variables from the observed data, this difference in sample size should not make much difference in practice (although large differences in sample size may lead to a loss of precision in estimates). In addition to this formal statistical disclosure control to remove unique replicates, we recommend that researchers perform a manual check of the data, using a few rows of the dataset, to ensure that ‘synthpop’ has not generated any data corresponding to real participants (this is primarily a sanity check, as the ‘sdc’ command should automatically remove all such cases).

The second factor to consider is whether the synthetic dataset successfully maintains relations between variables (although even synthetic datasets which do not maintain relations between variables can still be useful for reproducing code and checking for errors; ( Shepherd *et al.*, 2017)). The ‘synthpop’ package provides a suite of useful tools for comparing the synthesised data against the observed data. This includes a simple comparison of the distributions of each variable, through to more complex conditional associations, such as in a multivariable regression. Ideally, these should be similar between the observed and synthetic datasets, although random variation and imperfections in the synthesising process are of course inevitable. Formal measures of ‘utility’, comparing the synthetic to the observed data, are also available within the ‘synthpop’ package, but are not discussed here ( Raab *et al.*, 2017).

There are no definitive guidelines for successful synthesis, although some suggestions and recommendations can be made ( Nowok *et al.*, 2016; Raab *et al.*, 2017). First, when synthesising a large number of variables, tree-based methods are often quicker than parametric methods. Second, tree-based methods may reconstruct the observed data structure more faithfully than parametric methods. However, this advice may not hold in all circumstances, and we urge users to check the synthesised results against the observed data and examine whether the similarity is sufficient; if not, try a different specification and compare results. For instance, one can try using a parametric method, or an alternative tree-based method. In our experience, the order in which variables are synthesised can also influence the correspondence with the observed data, although not in all circumstances. While synthesising the data in any order may be sufficient, we have found that synthesising the exposure(s) and outcome(s) last sometimes maintains the relations between variables more faithfully, although this does somewhat contradict the advice given in ( Raab *et al.*, 2017), who recommend synthesising the most important variables first (although if the dataset is being synthesised for more general use and does not have specific exposures or outcomes, synthesising the variables in any order will likely suffice). Note that as synthetic data generation using ‘synthpop’ uses predictive modelling, it is not necessary to consider the causal structure of the data (i.e., the data-generating mechanisms) when synthesising data using this approach. Due to random variation, some synthetic datasets may be closer to the observed by chance; using a different starting seed may be another option to explore to improve the correspondence between synthetic and observed data. We advise that users test different synthesising methods, and see what works best for their specific dataset in terms of replicating the properties of the observed data (e.g., one method may maintain the relationship between the exposure and outcome more faithfully than another). For similar, but more detailed, guidelines on using ‘synthpop’, see ( Raab *et al.*, 2017). There are no definitive rules on what constitutes ‘successful’ synthesis, so again researchers must use their subjective judgement.

There may be a trade-off between the two aims introduced at the start of this section. For instance, methods of disclosure control may alter the relations between variables by removing some observations; conversely, a synthesised dataset which matches the observed data more faithfully may be at an increased risk of participant disclosure. Synthesis using parametric methods may reduce the number of unique replicates but at the same time result in less faithful synthetic datasets, while CART methods may better recreate the original data but result in more unique replicates, for example. Given the competing demands of creating a synthesised dataset close to the original vs reducing the risk of participant disclosure, there may be an iterative cycle between these potentially-conflicting requirements.

## ALSPAC guidelines for releasing synthetic data using ‘synthpop’

Here, we detail the guidelines required by all users of ALSPAC data wanting to make their synthesised data openly available. Please note, these are subject to change as the process develops over time. The most recent version of the ‘ ALSPAC synthetic data checklist’ will be available online. For many of these steps example code is provided below:

1) When submitting an ALSPAC proposal to access the data, make sure to state that you intend to release synthesised data. This can be noted by an amendment at a later date if necessary.
2) Reduce the number of variables and observations in the dataset you plan to synthesise to only those required to replicate the results in the paper (e.g., if the original dataset contains 15,645 observations and 20 variables, while the final analyses contain 4,000 observations and only use 10 variables, all additional observations and variables should be removed prior to synthesis). To avoid releasing a large number of synthetic variables, synthetic datasets should include fewer than 50 variables; if you need to synthesise more than 50 variables, please talk to ALSPAC and provide justification first.
3) Check whether there are individuals uniquely identified in both the observed and synthetic datasets. If there are, these should be removed from the synthetic dataset. Perform a manual check on a handful of cases as a sanity check to make sure there are no unique replicates in the observed and synthetic datasets.
4) Check that the distributions of all synthesised variables are similar to those in the observed data. Simply visualising the data (e.g., in bar charts or histograms) should provide this information (see worked example below).
5) Check that for key variables of interest (e.g., exposure and outcome) the relationships between synthesised variables are comparable to those in the observed data (e.g., via univariable and multivariable regressions). Or, in other words, re-estimate your substantive analysis.
6) Include a variable at the beginning of the synthetic dataset named ‘FALSE_DATA’, with values of ‘FALSE_DATA’ for all observations, to ensure it is clear that the dataset contains synthetic data, rather than real observations ( Nowok *et al.*, 2017).
7) Include a disclaimer in the published paper, and alongside the synthetic data, making it clear to users that the data are synthetic and should not be used for any subsequent research or publications. We recommend the following statement: “ *These are synthesised ALSPAC datasets, and are* not *suitable for research purposes. The relations between variables are unlikely to be maintained perfectly, so there is the risk when using these synthesised datasets that results may differ from the true data. Only the actual, observed, ALSPAC data should be used for formal research and analyses reported in published work.*”.
8) Provide the DOI or a suitable weblink as to where the synthetic data are stored (e.g., GitHub, Dryad, or Open Science Framework.
9) Agree to provide details of downloads/requests to use the synthetic data if ALSPAC request such information (if possible).
10) Agree to publish the script that developed the synthetic dataset to sit alongside the dataset (including code for all variable name changes and variable recodes and derivations from the original ALSPAC data, to facilitate reproducibility).

## Example ‘synthpop’ script

**Figure 1.**
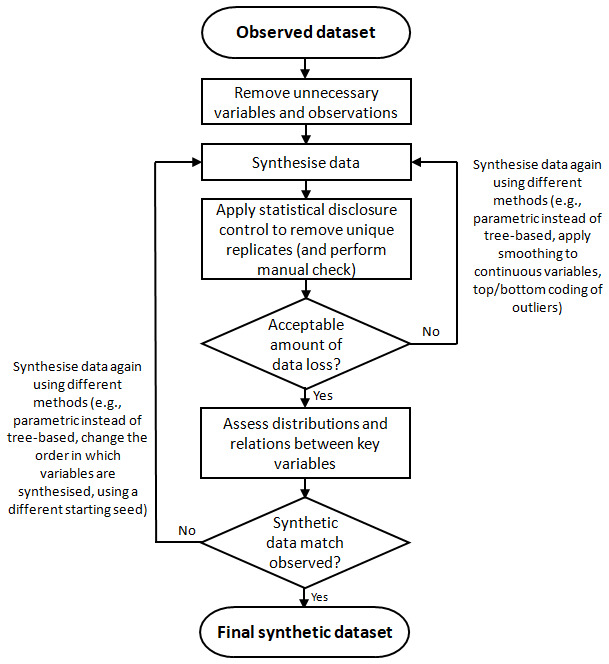
Flow-chart detailing the process for synthesising data to ensure that synthetic data is non-disclosive whilst maintaining relations between variables. Note that this may be an iterative process to find a synthetic dataset which meets these two potentially-competing demands. Note also that both ‘acceptable amounts of data loss’ and ‘synthetic data matching observed’ are subjective judgment calls.

In this section, we will detail the basics of how to synthesise ALSPAC data using the ‘synthpop’ package, and how to compare between observed vs synthesised datasets in a simple regression context. Figure 1 summarises this process. The example here is based on data from an openly available subset of the ALSPAC data ( Northstone *et al.*, 2022) ( https://osf.io/8sgze) so that readers can reproduce these steps. The substantive analysis is a logistic regression with the outcome being a diagnosis of depression in ALSPAC offspring at age 17 years (based on revised Clinical Interview Schedule; ( Lewis *et al.*, 1992)), and depression scores from mothers 8-months post-delivery as the exposure (using Edinburgh Postnatal Depression Scale; ( Cox *et al.*, 1987)), adjusting for a range of sociodemographic confounders (maternal age at delivery, maternal educational attainment, child gender, maternal home ownership status, and maternal ethnicity). This example was conducted using R version 4.0.4 ( R Development Core Team, 2021) and version 1.6-0 of the ‘synthpop’ package.

Step-by-step guide for synthesising data:

1. Install the ‘synthpop’ package in R and load it.
install.packages("synthpop")
library(synthpop)
2. Read in the observed dataset (here a Stata .dta file, using the R package ‘readstata13’), and then keep only variables and observations used in final analyses (note that some pre-processing of these variables has been omitted here; see the associated “SynthPopExample.R” script for full details).
dat <- read.dta13("Master_MSc_Data.dta")
dat <- dat[, c("gender", "bwt", "gest", "ethnic", "matage", "mated", "pated", "msoc", "psoc", "housing", "marital", "parity", "pregSize", "mat_dep", "depression_17")]
cca_marker <- complete.cases(dat[, c("gender", "ethnic", "matage", "mated", "housing", "mat_dep", "depression_17")])
dat <- dat[cca_marker == TRUE, ]
3. Take the observed dataset and synthesise using the ‘syn’ command (setting a seed so it is reproducible). The example here just uses the default ‘classification and regression trees’ method; for additional options, see ( Nowok *et al.*, 2016) and the “SynthPopExample.R” script associated with this paper.
dat_syn <- syn(dat, seed = 13327)
4. Next, apply ‘statistical disclosure control’ to remove individuals with unique combinations of variables in both the observed and synthesised data. Tabulate the number and percentage of such cases using the ‘replicated.uniques’ command, and then remove them from the synthesised dataset using the ‘sdc’ command with the ‘rm.replicated.uniques’ option. In this example of 3,727 observations, only 4 (0.11%) are unique replicates to be removed from the synthesised dataset.
replicated.uniques(dat_syn, dat)
dat_syn <- sdc(dat_syn, dat, rm.replicated.uniques = TRUE)
5. Perform a manual check on a handful of cases to ensure that the ‘sdc’ command above worked and that there are no replicated unique individuals in the final synthetic dataset.
# Create a dataset of unique observed individuals
dat_unique <- dat[!(duplicated(dat) | duplicated(dat, fromLast = TRUE)), ]

# Create a dataset of unique synthetic individuals
syn_unique <- dat_syn$syn[!(duplicated(dat_syn$syn) | duplicated(dat_syn$syn, fromLast = TRUE)), ]

# Select 10 rows at random from the unique observed dataset
row_unique <- dat_unique[sample(nrow(dat_unique), 10), ]

# Check there are no duplicated observations (this should equal ‘0’)
sum(duplicated(rbind.data.frame(syn_unique, row_unique)))
6. Compare the distribution of variables between the observed and synthetic datasets using the ‘compare’ command. This provides a series of descriptive tables and figures (see Figure 2), which compare the marginal distribution of all variables in the synthesised and observed datasets. Figure 2 illustrates that the synthetic data matches the observed distribution of each variable very well (including NAs/missing values).
compare(dat_syn, dat, stat = “count”, nrow = 3, ncol = 5)
7. Run an unadjusted model comparing the association between the exposure and outcome in both observed and synthetic datasets (here, the exposure is the ALSPAC mother’s depressive symptoms score 8-months post-delivery, and the outcome is whether their offspring was depressed at age 17 years). First store a model using the synthetic data (using the ‘glm.synds’ command), and then compare this against a model using the actual data. In our example, the association in the synthetic data is similar, but slightly larger, to that in the observed data ( Figure 3; note that, by default, ‘synthpop’ converts all coefficients to *z*-values so that all coefficients are on the same scale and hence easier to compare).
model.syn <- glm.synds(depression_17 ~ mat_dep, family = “binomial”, data = dat_syn)
compare(model.syn, dat)
8. Repeat step 7, using a multivariable model adjusting for additional covariates (i.e., our substantive analysis model). Here, we can see that the relations between the outcome and other variables are also similar, although not exactly the same, in both observed and synthetic datasets ( Figure 4; we also stress here that in a causal analysis between the exposure and outcome the coefficients of these additional covariates do not have a straightforward interpretation [see Westreich & Greenland, 2013 on the ‘table 2’ fallacy]).
model.syn2 <- glm.synds(depression_17 ~ mat_dep + matage + ethnic + gender + mated + housing, family = “binomial”, data = dat_syn) compare(model.syn2, dat)
9. Repeat steps 3 to 8 until you are happy that: a) the number of unique replicates removed from the synthetic dataset is sufficiently low; and b) the distributions and relations between variables in the observed and synthetic datasets are sufficiently similar. Note that both of these decisions are subjective judgements.
10. Add a variable called ‘FALSE_DATA’, with the value of ‘FALSE_DATA’ for all observations, to the start of the synthetic dataset, so users know that the dataset contains synthetic – as opposed to observed – data.
dat_syn$syn <- cbind(FALSE_DATA = rep(“FALSE_DATA”, nrow(dat_syn$syn)), dat_syn$syn)
11. Save the synthesised dataset. In our example, we have saved the synthesised data as R, CSV and Stata data files, respectively.
write.syn(dat_syn, file = “syntheticData”, filetype = “Rdata”)
write.syn(dat_syn, file = “syntheticData”, filetype = “csv”)
write.syn(dat_syn, file = “syntheticData”, filetype = “Stata”, convert.factors = “labels”)

**Figure 2.**
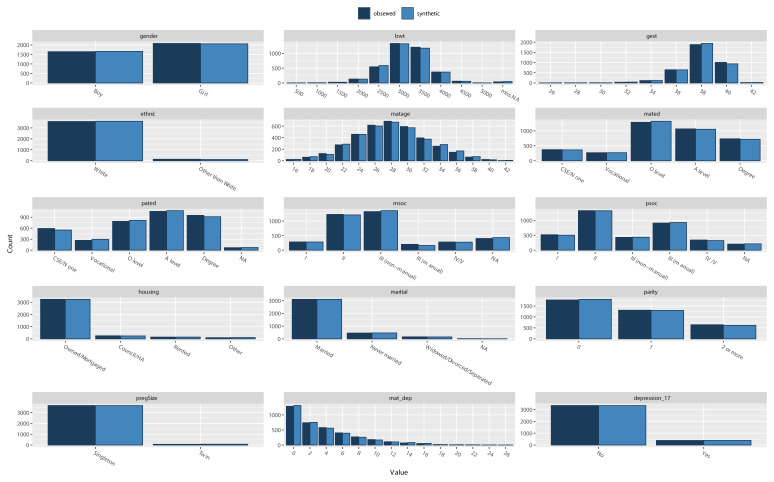
Comparing variable distributions in observed (dark blue) and synthetic (light blue) datasets.

**Figure 3.**
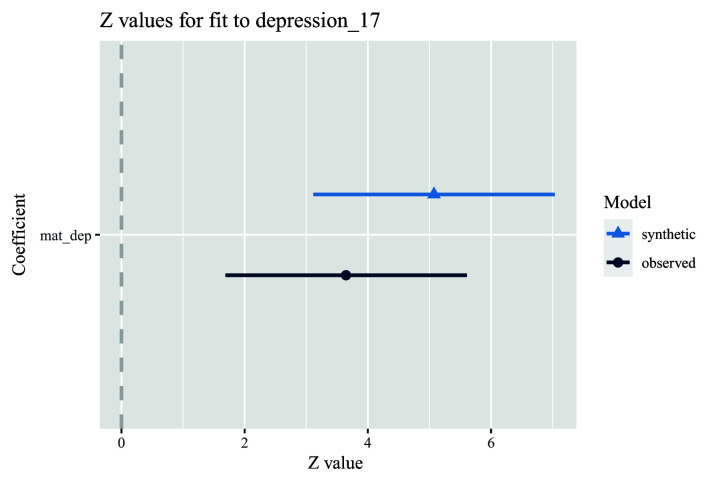
Example analysis comparing univariable associations between an exposure (maternal depressive symptoms score; mat_dep) and an outcome (offspring depression diagnosis; depression_17) in both observed (dark blue) and synthetic (light blue) datasets. Note also that the results in the plot are on a z-value scale.

**Figure 4.**
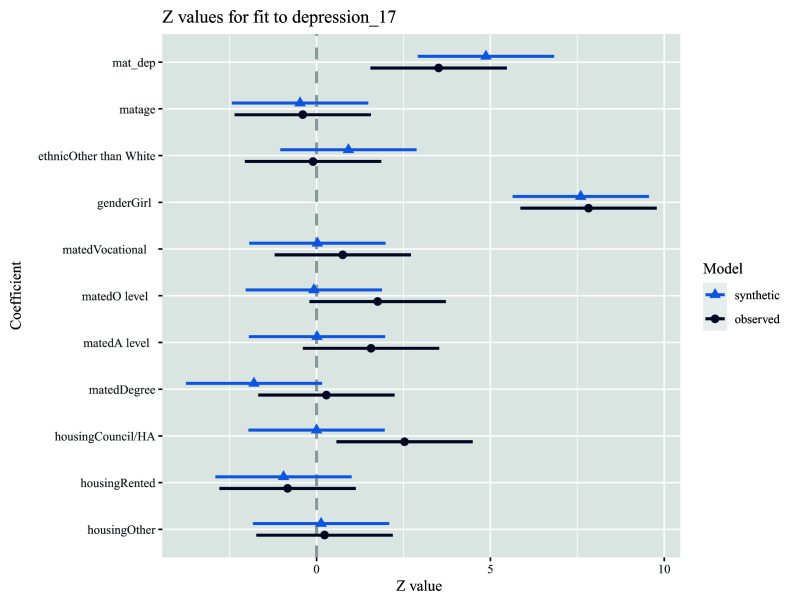
Example analysis comparing multivariable associations between an exposure (maternal depressive symptoms score; mat_dep) and an outcome (offspring depression diagnosis; depression_17) in both observed (dark blue) and synthetic (light blue) datasets. Note also that the results in the plot are on a z-value scale.

## Applied longitudinal example – using ‘synthpop’ as an educational and open research tool

The above example highlights how simulated data using ‘synthpop’ can mimic basic results from longitudinal studies. However, one of the key assets of longitudinal studies like ALSPAC is the ability to capture traits over time in a repeated measures context. Currently, ‘synthpop’ does not have a way to test this within the package. However, using the framework above, it is possible to simulate repeated measures (for example, but not limited to, height, weight, substance use, test scores and mental health) to create trajectories of simulated data that mimic the real data. Here we give two examples adapted from existing research within ALSPAC to highlight how repeated measures data can be simulated using ‘synthpop’ (note that, unlike the simple example above, the observed ALSPAC data for these analyses are not openly-available, although the synthetic data are; see the 'Data Availability' section). For both examples, we synthesised datasets using the default CART approach with these longitudinal data in ‘wide’ format (i.e., one row per participant with time-points as separate variables); synthesised data were then converted to ‘long’ format for the multi-level modelling analysis (i.e., multiple observations per participant with one row per time-point).

The first example examines height trajectories from childhood to early adulthood using a multilevel modelling framework, similar to that of Howe *et al.* ( Howe *et al.*, 2012; Howe *et al.*, 2016), using up to eight occasions of height between approximately 7 to 18 years of age. The second example uses growth mixture modelling to examine associations between adolescent self-esteem and depression trajectories across adolescence and early adulthood, and then depression trajectories across adolescence and early adulthood associated with later depression, similar to that of Kwong *et al.* ( Kwong *et al.*, 2019) and López-López *et al.* ( López-López *et al.*, 2020), using up to nine occasions of depressive symptoms between approximately 10 and 24 years of age. For further details on these methods, please refer to these original papers.

**Figure 5.**
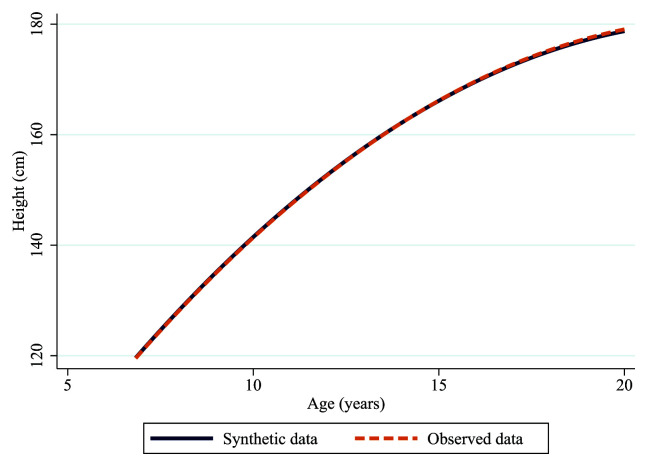
Synthetic (solid blue line) and observed (dashed orange line) trajectories of height in ALSPAC.

As shown in Figure 5 and Table 1, synthetic height trajectories perform almost identically to the observed data when assessed using multi-level growth models. Both trajectories show the same rate of change and the estimates from the model are nearly identical across both datasets. The marginal differences are likely to reflect different sample sizes or random variability in the synthesis model (synthetic n=10,261, observed=10,059).

**Table 1.** Synthetic and observed estimates from the height trajectories.

	Observed ( *n*=10,059, *n _obs_* =53,853)				Synthetic ( *n*=10,261, *n _obs_* =53,864)			
Fixed effects	*Beta*	*Std Err*	*95% CI*	*P*	*Beta*	*Std Err*	*95% CI*	*P*
Age	5.19	0.01	5.17 to 5.21	<0.001	5.17	0.01	5.15 to 5.19	<0.001
Age ^2^	-0.24	0.00	-0.24 to -0.23	<0.001	-0.24	0.00	-0.25 to -0.24	<0.001
Intercept	152.74	0.07	152.60 to 152.88	<0.001	152.80	0.07	152.66 to 152.94	<0.001
Random effects	*Estimate*	*Std Err*	*95% CI*		*Estimate*	*Std Err*	*95% CI*	
var(Age)	0.45	0.01	0.43 to 0.47		0.48	0.01	0.46 to 0.51	
var(Age ^2^)	0.03	0.00	0.03 to 0.04		0.03	0.00	0.03 to 0.03	
var(Intercept)	46.54	0.72	45.16 to 47.97		45.50	0.70	44.14 to 46.90	
cov(Age, Age ^2^)	0.09	0.00	0.09 to 1.00		0.09	0.00	0.09 to 1.00	
cov(Age, Intercept)	0.95	0.07	0.82 to 1.074		0.96	0.07	0.82 to 1.09	
cov(Age ^2^, Intercept)	-0.51	0.02	-0.55 to -0.47		-0.48	0.02	-0.52 to -0.45	
var(Residual)	6.32	0.05	6.22 to 6.43		7.35	0.06	7.23 to 7.47	

Note: a random intercept and random slope model was used to estimate the height trajectories, mean centering the age variable to 12 years (the mean age of all the age variables). We used a quadratic polynomial age term to allow for non-linearity. var: variance; cov: covariance; Std Error: standard error; CI: Confidence interval

**Figure 6.**
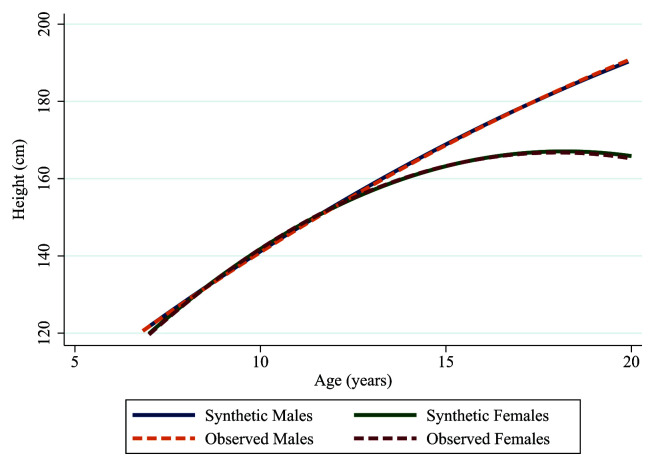
Synthetic male (solid blue line), synthetic female (solid green line), observed male (dashed orange line) and observed female (dashed red line) trajectories of height in ALSPAC.

Furthermore, when adding an interaction term of sex to estimate male and female height trajectories, the synthetic and observed data created trajectories that were nearly identical, as shown in Figure 6 and Table 2. However, the main effect of sex (i.e., the difference in height between males and females at age 12 [the mean age of all the assessments]) did vary between the observed and synthetic data, which is likely a result of random variability in the synthesis model. However, as shown in Figure 6, this had little effect on the estimation of the height trajectories and it is worth noting the confidence intervals for the main effect overlap as in the example above.

**Table 2.** Synthetic and observed estimates from the height trajectories by sex.

	Observed ( *n*=10,053, *n _obs_* =53,818)				Synthetic ( *n*=9,834, *n _obs_* =52,557)			
Fixed effects	*Beta*	*Std Err*	*95% CI*	*P*	*Beta*	*Std Err*	*95% CI*	*P*
Age	5.65	0.01	5.64 to 5.67	<0.001	5.65	0.01	5.63 to 5.67	<0.001
Female	0.05	0.14	-0.23 to 0.33	0.751	-0.35	0.14	-0.63 to -0.06	0.016
Female*Age	-0.98	0.01	-1.01 to -0.96	<0.001	-0.99	0.01	-1.02 to -0.97	<0.001
Age ^2^	-0.11	0.00	-0.11 to -0.10	<0.001	-0.12	0.00	-0.12 to -0.11	<0.001
Female*Age ^2^	-0.28	0.00	-0.29 to -0.27	<0.001	-0.26	0.00	-0.27 to -0.25	<0.001
Intercept	152.72	0.10	152.53 to 152.92	<0.001	153.00	0.10	152.80 to 153.19	<0.001
Random effects	*Estimate*	*Std Err*	*95% CI*		*Estimate*	*Std Err*	*95% CI*	
var(Age)	0.14	0.01	0.13 to 0.15		0.17	0.01	0.16 to 0.18	
var(Age ^2^)	0.01	0.00	0.01 to 0.01		0.01	0.00	0.01 to 0.01	
var(Intercept)	46.50	0.72	45.12 to 47.93		45.20	0.71	43.83 to 46.62	
cov(Age, Age ^2^)	0.01	0.00	0.01 to 0.01		0.02	0.00	0.01 to 0.02	
cov(Age, Intercept)	0.89	0.04	0.80 to 0.97		0.77	0.05	0.68 to 0.87	
cov(Age ^2^, Intercept)	-0.53	0.01	-0.55 to -0.50		-0.53	0.01	-0.55 to -0.50	
var(Residual)	6.54	0.06	6.44 6.65		7.54	0.06	7.42 to 7.67	

Note: a random intercept and random slope model was used to estimate the height trajectories, mean centering the age variable to 12 years (the mean age of all the age variables). We used a quadratic polynomial model to allow for non-linearity. Female was coded as 0=males, 1=females. var: variance; cov: covariance; Std Error: standard error; CI: Confidence interval

**Figure 7.**
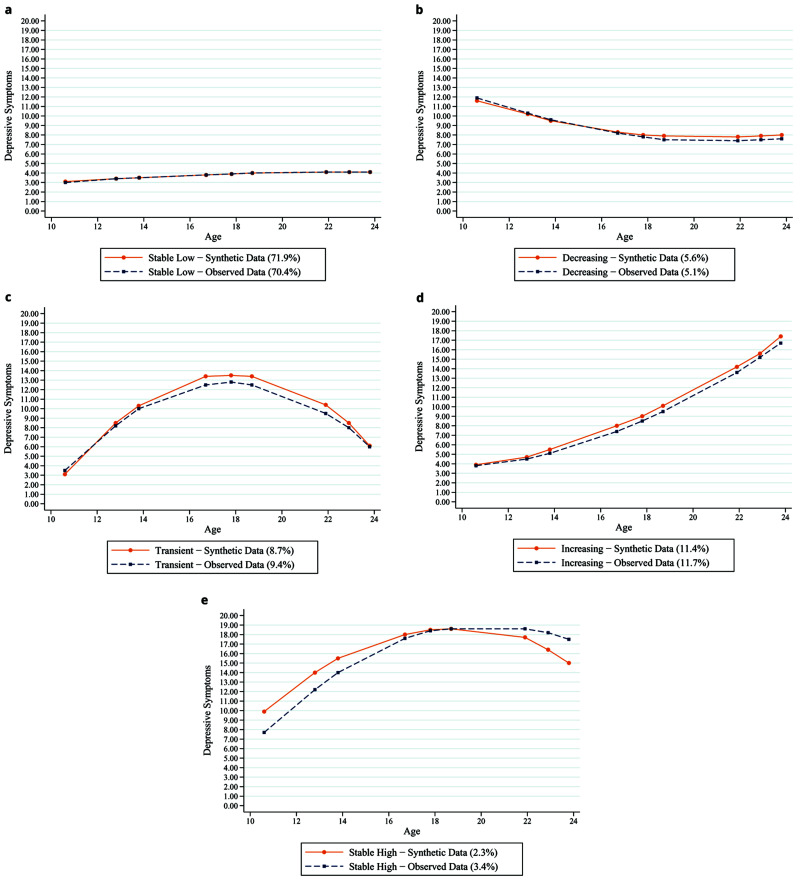
Synthetic (dashed blue lines) and observed (solid orange lines) data depression trajectories using growth mixture modelling.

Building on the example above, we show that synthetic data can also be used for more advanced forms of growth curve modelling. As shown in Figure 7, synthetic datasets produce similar patterns of observed depression trajectories when analysed using growth mixture modelling (GMM). Several features used to assess model fit within GMM were similar between the synthetic and observed data, including entropy, Bayesian Information Criterion (BIC) and Sample Size Adjusted BIC, which were 0.735, 249378.07 and 249304.98 for the synthetic data and 0.734, 251430.95 and 251357.86 for the observed data, respectively. In addition, the estimates from adjusted regression models using synthetic data mimic the estimates from the adjusted regression models using observed data. For example, Table 3 shows that higher self-esteem in childhood is associated with lower relative risk ratios for each of the trajectories, and this matches across both the synthetic data and the observed data. Furthermore, Table 4 shows that worse depression trajectories across adolescence and early adulthood are associated with greater odds of depression later on, and these estimates are almost identical for both the synthetic and observed datasets.

**Table 3.** Association between self-esteem and different depression trajectories (RRR, Std Err and P value) with synthetic and observed data.

		Stable-Low vs Stable High	Stable Low vs Increasing	Stable Low vs Transient	Stable Low vs Decreasing
Synthetic n=3,719	Self-esteem	0.17 (0.02), P<0.001	0.44 (0.03), P<0.001	0.29 (0.02), P<0.001	0.51 (0.04), P<0.001
Observed n=3,850	Self-esteem	0.11 (0.01), P<0.001	0.42 (0.03), P<0.001	0.23 (0.02), P<0.001	0.58 (0.05), P<0.001

Note: Depression trajectories were created used growth mixture modelling using 9 time points (see ( Kwong *et al.*, 2019) for further details). Models adjusted for maternal depression, maternal education and financial problems. Self-esteem was standardised to have a mean of 0 and a SD of 1. Higher self-esteem scores reflect higher self-esteem. RRR: relative risk ratio; Std Err: standard error.

**Table 4.** Association between depression trajectories and later depression using synthetic and observed data.

	Observed ( *n*=3,432)				Synthetic ( *n*=3,215)			
*Comparison*	*OR*	*Std Err*	*95% CI*	*P*	*OR*	*Std Err*	*95% CI*	*P*
Stable-Low vs Stable High	9.61	2.07	6.32 to 14.63	<0.001	9.18	2.48	5.40 to 15.59	<0.001
Stable Low vs Increasing	5.78	0.71	4.53 to 7.36	<0.001	5.27	0.65	4.12 to 6.71	<0.001
Stable Low vs Transient	3.50	0.46	2.70 to 4.52	<0.001	2.75	0.38	2.09 to 3.62	<0.001
Stable Low vs Decreasing	2.46	0.49	1.67 to 3.62	<0.001	3.33	0.63	2.30 to 4.81	<0.001

Note: Depression trajectories were created used growth mixture modelling using 9 time points (see ( Kwong *et al.*, 2019) for further details). Models adjusted for maternal depression, maternal education and financial problems. Later depression was assessed two years after the trajectories. OR: odds ratio; Std Err: standard error; CI: confidence interval.

## Discussion

We recognise the importance of open science practices for longitudinal population studies, while also acknowledging the need for such studies to maintain control over access to potentially-sensitive data. We believe that the synthetic data approach described in this paper provides a reasonable compromise between these competing demands, allowing data users to make de-identified synthesised data openly available while complying with data security and participant confidentiality best practice (see also; ( Quintana, 2020; Shepherd *et al.*, 2017)). While the focus of this paper has been on longitudinal studies, and ALSPAC in particular, the suggestions and guidelines in this paper may also help inform the sharing of potentially-sensitive individual-level data in many other areas and disciplines ( Quintana, 2020). These methods can also be used to construct synthetic datasets for educational purposes, such as demonstrating complex methods such as using repeated measures for generating trajectories and growth mixture modelling, as illustrated above. We end with a brief discussion on some clarifications and potential limitations of this synthetic data approach.

While undoubtedly a useful pedagogical tool, and beneficial for open science practices, we state clearly here that these synthetic datasets should *not* be used in place of the actual observed data for research purposes; that is, synthesised data should never be used for a final published analysis. While hopefully similar on average to the observed data, the synthesised relations between variables may not be preserved perfectly and hence may provide different results, so published work should only ever be based on the observed data. To try and avoid synthetic ALSPAC data being used for research purposes – whether knowingly or otherwise – we have put a number of safe-guards in place (see points 2, 6 and 7 of the ‘ALSPAC guidelines for releasing synthetic data using ‘synthpop’’ section). Additional measures will be put in place should synthetic ALSPAC data be found to have been used for these purposes.

We also note that the recommendations and guidelines above only apply to data synthesised using ‘synthpop’, rather than via other simulation methods, such as using ALSPAC summary statistics or regression results to inform simulation parameters (for an example study using the latter approach to explore selection bias in ALSPAC, see ( Millard *et al.*, 2023)). We make this distinction because these latter forms of simulation are only based on summary-level data, meaning that simulated data points do not correspond to data from individuals in the observed dataset and can sometimes take on impossible values (e.g., negative and/or decimal values, if the original scale was positive and/or only took integer values). In contrast, when using sampling, tree-based methods or ranked modelling methods within ‘synthpop’, the synthesised values are taken directly from the observed data, making synthesised data more faithful to the observed data, but also potentially increasing the risk of disclosure. In addition, the equations and parameters used to simulate data from summary statistics are transparent, meaning that it is obvious that the data are simulated. For ‘synthpop’, on the other hand, the synthesis methods and parameters are much more opaque; greater attention to potential disclosure of individual-level information is therefore needed when synthesising data using ‘synthpop’.

The present paper has focused on the package ‘synthpop’, although other software for synthetic data generation are available ( Raghunathan, 2021; Shepherd *et al.*, 2017), such as Imputation and Variance Estimation Software ( IVWare; ( Raghunathan *et al.*, 2022)). However, at present, for synthesising ALSPAC data we recommend using the ‘synthpop’ package because this contains built-in statistical disclosure control functionality to automatically remove potentially disclosive observations. Please talk to ALSPAC first if you wish to use an alternative method for data synthesis. At present, ALSPAC also does not permit data to be made openly-available via other approaches which aim to anonymise and de-identify participants (e.g., statistical disclosure control; Templ *et al.*, 2015) as these still largely make use of observed – rather than wholly synthetic – data, although this may change in the future.

A potential limitation of the ‘synthpop’ package is that is primarily designed for datasets with independent observations, not more complex situations such as multi-level/hierarchical data. While it can be applied in such circumstances – such as for longitudinal modelling with repeated data, as demonstrated above – the correspondence between the observed and synthetic data needs to be assessed carefully and cannot be assumed to hold. For instance, when using the standard ‘synthpop’ package, synthesising data in ‘wide’ format (as used here) appears to work well, but is unlikely to hold for data in ‘long’ format as information on the relations between observations within individuals would be lost. There are user-written extensions to synthpop which describe how to synthesise hierarchical data (see http://gradientdescending.com/synthesising-multiple-linked-data-sets-in-r/), but these methods are not covered in this paper. The ‘synthpop’ package can, however, be used to synthesise other data types, such as time-to-event/survival data (see also ( Smith *et al.*, 2022)).

A further limitation is that the ‘synthpop’ package is primarily only available in the R programming language (although an implementation of ' synthpop' is available in Python;). While alternative synthesis software such as IVEware are compatible with a larger number of statistical programmes (e.g., R, Stata, SPSS and SAS), as discussed above we do not recommend this approach due to a lack of statistical disclosure control measures. We hope the step-by-step guide, along with the more detailed R scripts associated with this data note, will enable even researchers unfamiliar with the R programming language to successfully create synthesised datasets.

The release of synthetic datasets from longitudinal population-based studies is novel (to the best of our knowledge ALSPAC is the first such study to create specific guidelines for sharing synthetic datasets). We would welcome other longitudinal population-based studies to build upon the knowledge and guidelines developed by ALSPAC in creating their own synthetic data sharing policies, furthering the promotion of open and transparent research. We believe that our current approach is comprehensive and therefore our guidelines could be readily adopted by other studies and adapted as required. We would also welcome feedback from other longitudinal population-based studies and their users to help us improve our guidelines and processes.

There are also some challenges and open questions, for instance, whether potential safe-guards to prevent synthetic data being used in the place of real data (as discussed above). A further issue is the question of ownership; that is, who owns the synthetic data? At present, synthetic ALSPAC data is allowed to be made freely-available to all without the need for managed access or agreements to be signed. This is largely to make the synthetic data and open and accessible as possible, while minimising additional administrative work for ALSPAC staff. If needed, however, this may change in the future. An additional question is whether such synthetic datasets meet journal and funder requirements for data sharing. Although sharing synthetic data is clearly better than sharing no data, clarity from funders and journals is required to definitively answer this. However, in our experience some journals that have clear policies mandating data sharing have been open to the sharing of synthetic ALSPAC data, given that the raw ALSPAC data cannot be released.

To end, we stress that, wherever possible, the observed raw data – alongside the analysis code ( Goldacre *et al.*, 2019; Goldstein *et al.*, 2020; Localio *et al.*, 2018) – should be made openly available to facilitate fully-reproducible open science ( Goldstein, 2018; Harper, 2019; Munafò *et al.*, 2017; Peng *et al.*, 2006). Where this is not feasible, either to preserve participant confidentiality or to ensure only legitimate researchers can access the resource, releasing synthetic datasets is a useful and pragmatic alternative, which enables research to be ‘quasi-reproducible’ ( Shepherd *et al.*, 2017). For a recent example of such an approach which includes openly-available synthetic ALSPAC data, see ( Major-Smith, 2023). We hope to see an increasing number of papers, both in ALSPAC and more widely, using synthetic generation methods to make potentially-sensitive datasets openly available.

## Consent

Ethical approval for this study was obtained from the ALSPAC Ethics and Law Committee and the Local Research Ethics Committees. Informed consent for the use of data collected via questionnaires and clinics was obtained from participants following the recommendations of the ALSPAC Ethics and Law Committee at the time. Study participants have the right to withdraw their consent for elements of the study or from the study entirely. Full details of the ALSPAC consent procedures are available on the study website ( http://www.bristol.ac.uk/alspac/researchers/research-ethics/).

## Data Availability

Please see the ALSPAC data management plan which describes the policy regarding data sharing ( http://www.bristol.ac.uk/alspac/researchers/data-access/documents/alspac-data-management-plan.pdf), which is by a system of managed open access. Other than the freely available ALSPAC dataset and the synthetic datasets (see below), all other data used for this submission will be made available on request to the Executive ( alspac-exec@bristol.ac.uk). These datasets are linked to ALSPAC project number B4301, please quote this project number during your application. The following datasets and analysis code files supporting this submission are available on DM-S’s GitHub page ( https://github.com/djsmith-90/synthetic-data, available under a GPL-3.0 license, archived at the time of publication: https://doi.org/10.5281/zenodo.10457847, djsmith-90, (2024)) ; this includes: “SynthPopExample.r”: An example R script to replicate the step-by-step example using the openly available ALSPAC dataset, as well as explore some additional ‘synthpop’ functionality (parametric synthesis, smoothing parameters, top- and bottom-coding, synthesis using different numbers of variables, etc.). The openly available subset of ALSPAC data used for this example is available here ( https://doi.org/10.17605/OSF.IO/8SGZE; Northstone *et al.*, 2022). “synthpop_repeated-measures_script”: This script processes and synthesises the datasets for the longitudinal modelling examples. “Simulated_height.dta”: The synthesised ALSPAC dataset for the multi-level growth models of height, in Stata format (note that the corresponding observed ALSPAC data files are not available for these analyses). “analysis_height”: The Stata script to perform the multi-level growth models on the “Simulated_height.dta” dataset. “Simulated_depression_mplus.dta”: The synthesised ALSPAC dataset for performing the growth mixture modelling analysis of depression, in Stata format (note that the corresponding observed ALSPAC data files are not available for these analyses). “prep_analysis_depression”: Script which initially processes the data for the growth mixture modelling analysis (in Stata), followed by the MPlus code to perform the growth mixture modelling analysis. The steps below highlight how to apply for access to the data included in the data note and all other ALSPAC data: Please read the ALSPAC access policy ( http://www.bristol.ac.uk/media-library/sites/alspac/documents/researchers/data-access/ALSPAC_Access_Policy.pdf) which describes the process of accessing the data and samples in detail, and outlines the costs associated with doing so. You may also find it useful to browse our fully searchable research proposals database ( https://proposals.epi.bristol.ac.uk/?q=proposalSummaries), which lists all research projects that have been approved since April 2011. Please submit your research proposal ( https://proposals.epi.bristol.ac.uk/) for consideration by the ALSPAC Executive Committee. You will receive a response within 10 working days to advise you whether your proposal has been approved. Please note that the study website contains details of all the data that is available through a fully searchable data dictionary and variable search tool: http://www.bristol.ac.uk/alspac/researchers/our-data/.
