# Supplementary material for: Releasing synthetic data from the Avon Longitudinal Study of Parents and Children (ALSPAC): Guidelines and applied examples
Source: Wellcome Open Res. 2024 Dec 24;9:57. Originally published 2024 Feb 19. [Version 2] doi: 10.12688/wellcomeopenres.20530.2 (PMC11809151; doi:10.12688/wellcomeopenres.20530.2)
Supplement: Peer review history

# Reviewer report: Reviewer response for version 2

**DOI:** 10.21956/wellcomeopenres.25979.r116323

**Published:** 2025-02-05

**Authors:** Phillip Melton

**Affiliations:** 1 University of Tasmania, Hobart, Tasmania, Australia

I thank the authors of this manuscript for their thoughtful responses to my earlier comments that they have addressed in this updated version of the manuscript.

Is the rationale for developing the new method (or application) clearly explained?

Yes

Is the description of the method technically sound?

Partly

Are the conclusions about the method and its performance adequately supported by the findings presented in the article?

Yes

If any results are presented, are all the source data underlying the results available to ensure full reproducibility?

Yes

Are sufficient details provided to allow replication of the method development and its use by others?

Yes

Reviewer Expertise:

biostatisics, longitudinal data, cohort studies, data sciene

I confirm that I have read this submission and believe that I have an appropriate level of expertise to confirm that it is of an acceptable scientific standard.

# Reviewer report: Reviewer response for version 2

**DOI:** 10.21956/wellcomeopenres.25979.r116944

**Published:** 2025-01-10

**Authors:** Venet Osmani

**Affiliations:** 1 Queen Mary University of London, London,, UK

The motivation of this paper is commendable. There is indeed an urgent need to make health data more widely available to advance science and mitigate reproducibility crisis.

 However, there are a number of ways the paper can be further improved.

1. The authors' goal, stated in the introduction, is to generate research-quality synthetic data, i.e. data where 'any analyses and conclusions ought to be similar'. However, the method (synthpop) is not very well suited for this objective. Indeed, the authors state this in the discussion that synthetic variables may take on impossible values.
  Therefore, there appears to be little gain in using synthpop with respect to the approach adopted by the NHS artificial data pilot, where the risk of disclosure is virtually zero. Can authors comment on this point?
  Perhaps a more sophisticated method that can generate research-quality synthetic data should be considered if this goal is to be achieved.
2. Tree-based methods are not well equipped to handle longitudinal data. Authors address this issue by creating a 'wide' data format however at the expense of assumption that each time-point measurement is an independent variable, which of course is not correct.
3. I liked the recommendations. I would add another: to consider whether the synthetic dataset is useful, that is to compare whether results in a downstream task (e.g. an outcome prediction) are similar between the real and the synthetic datasets.

Is the rationale for developing the new method (or application) clearly explained?

Yes

Is the description of the method technically sound?

Yes

Are the conclusions about the method and its performance adequately supported by the findings presented in the article?

Partly

If any results are presented, are all the source data underlying the results available to ensure full reproducibility?

Yes

Are sufficient details provided to allow replication of the method development and its use by others?

Yes

Reviewer Expertise:

Machine learning

I confirm that I have read this submission and believe that I have an appropriate level of expertise to confirm that it is of an acceptable scientific standard, however I have significant reservations, as outlined above.

# Reviewer report: Reviewer response for version 2

**DOI:** 10.21956/wellcomeopenres.25979.r116321

**Published:** 2025-01-08

**Authors:** Sam Harper

**Affiliations:** 1 Department of Epidemiology, Biostatistics and Occupational Health, School of Population and Global Health, McGill University, Montreal, Canada

Overall, I think the authors have done a good job of responding to the comments of the reviewers, and at this point I think we can probably just agree to disagree regarding certain questions. Specifically, I still think it would help readers and those wanting to use 'synthpop' if the authors could provide at least some guidance about how to actually determine the 'faithfulness' of the synthetic vs. original datasets.

 The authors say:

 > *even datasets which randomly synthesise each variable independent of all others can still be useful to explore the underlying code/analysis approach and checking for errors, even if all relations between the variables are lost*

 But what kind of errors would I check for to determine whether or not the synthetic data are 'useful'? Just that the code can run, even if the results are not sensible? Or if X1 and X2 are correlated at 0.7 in the original source but the synthpop correlation is 0.1, is that useful? Again, I don't want to let the perfect be the enemy of the good here, but I even think that if the authors provided 1 or 2 examples of cases where they actually thought this *was not* useful, that would help readers a bit more. Given that they say, " we urge users to check the synthesised results against the observed data and examine whether the similarity is sufficient; if not, try a different specification and compare results". At least an example of 'non-sufficiency' here would help. 

 And I could not tell whether the authors specifically mention it in the paper, but I think that one of the other benefits of 'synthpop' could be to provide a complete replication package for a published paper when the underlying data are sensitive/confidential and cannot be released. Including the synthetic data in a complete set of scripts can be extraordinarily helpful for readers and reviewers to have a better understanding of exactly how researchers generated their estimates and figures, even if the underlying data must be synthetic. 

 Apart from that, I think the revised version is solid and will be helpful for readers interested in pushing open science further.

Is the rationale for developing the new method (or application) clearly explained?

Yes

Is the description of the method technically sound?

Partly

Are the conclusions about the method and its performance adequately supported by the findings presented in the article?

Yes

If any results are presented, are all the source data underlying the results available to ensure full reproducibility?

Yes

Are sufficient details provided to allow replication of the method development and its use by others?

Yes

Reviewer Expertise:

epidemiology, impact evaluation, reproducible research

I confirm that I have read this submission and believe that I have an appropriate level of expertise to confirm that it is of an acceptable scientific standard.

# Reviewer report: Reviewer response for version 2

**DOI:** 10.21956/wellcomeopenres.25979.r116322

**Published:** 2024-12-31

**Authors:** Neal Goldstein

**Affiliations:** 1 School of Public Health, Drexel University, Philadelphia, Pennsylvania, USA

I thank the authors for their responsiveness to my initial comments. I have no additional requests at this time.

Is the rationale for developing the new method (or application) clearly explained?

Partly

Is the description of the method technically sound?

Yes

Are the conclusions about the method and its performance adequately supported by the findings presented in the article?

Yes

If any results are presented, are all the source data underlying the results available to ensure full reproducibility?

Yes

Are sufficient details provided to allow replication of the method development and its use by others?

Yes

Reviewer Expertise:

Epidemiology

I confirm that I have read this submission and believe that I have an appropriate level of expertise to confirm that it is of an acceptable scientific standard.

# Reviewer report: Reviewer response for version 1

**DOI:** 10.21956/wellcomeopenres.22725.r87781

**Published:** 2024-09-11

**Authors:** Phillip Melton

**Affiliations:** 1 University of Tasmania, Hobart, Tasmania, Australia

The paper “Releasing synthetic data from the Avon the paper “Releasing synthetic data from the Avon Longitudinal Study of Parents and Children (ALSPAC): Guidelines and applied examples” by Major-Smith *et al*. provides a real-world example of how to produce synthetic data from a large population cohort. The authors provide examples of the methodology used in the R-package, synthpop, and then give an example of doing longitudinal analysis. Overall, the paper is well structured, but I have a few comments and suggestions that I hope the authors may find useful.

**General comments:**

- The discussion would benefit from a paragraph on recommendations for other cohort studies that may be considering developing guidelines around synthetic data. Several other longitudinal cohort studies would potentially benefit from this.
- Also, who technically owns the synthetic dataset is this the author or what role does ALSPAC play in this? Is there an agreement that the authors sign, and how is access managed?
- Would synthetic data meet EU GDPR data sharing guidelines, while it is artificial but may still apply (see Beduschi A *et al*.(2024 ^1^))  

**Specific comments:**

- P3: When mentioning other tree-based and parametric methods beyond CART, it would help to specify some of these.  
- P4. For longitudinal data how is potential disclosure managed because you now have temporal data that has the potential to identify participants?
- P5. More details on the specifics of the similarity of distributions. Guidance here or recommendations would be useful.
- Figure 4: Why are so many of the Z-values 0 for the synthetic data? Is this because they are potentially categorical?
- Tables: Showing the synthetic data and the observed data for each covariate on the same row may be easier to interpret for the reader.

Is the rationale for developing the new method (or application) clearly explained?

Yes

Is the description of the method technically sound?

Partly

Are the conclusions about the method and its performance adequately supported by the findings presented in the article?

Yes

If any results are presented, are all the source data underlying the results available to ensure full reproducibility?

Yes

Are sufficient details provided to allow replication of the method development and its use by others?

Yes

Reviewer Expertise:

biostatisics, longitudinal data, cohort studies, data sciene

I confirm that I have read this submission and believe that I have an appropriate level of expertise to confirm that it is of an acceptable scientific standard, however I have significant reservations, as outlined above.

## References

[1] : Synthetic data protection: Towards a paradigm change in data regulation?. *Big Data & Society* .2024;11(1) : 10.1177/20539517241231277 10.1177/20539517241231277

# Response

**Published:** 2024-12-17

**Authors:** Daniel Smith

**Affiliations:** University of Bristol, UK

*We thank the reviewer for their positive review and their constructive comments. We have responded to the reviewer’s comments in turn below, with the reviewer’s original comments in standard font and our responses in italics.*  

**General comments:**

 The discussion would benefit from a paragraph on recommendations for other cohort studies that may be considering developing guidelines around synthetic data. Several other longitudinal cohort studies would potentially benefit from this.

**Response​​​​​​​:** *This is a nice idea, thanks for suggesting it. We have now added a paragraph on this in the Discussion: “The release of synthetic datasets from longitudinal population-based studies is novel (to the best of our knowledge ALSPAC is the first such study to create specific guidelines for sharing synthetic datasets). We would welcome other longitudinal population-based studies to build upon the knowledge and guidelines developed by ALSPAC in creating their own synthetic data sharing policies, furthering the promotion of open and transparent research. We believe that our current approach is comprehensive and therefore our guidelines could be readily adopted by other studies and adapted as required. We would also welcome feedback from other longitudinal population-based studies and their users to help us improve our guidelines and processes.”*

 Also, who technically owns the synthetic dataset is this the author or what role does ALSPAC play in this? Is there an agreement that the authors sign, and how is access managed? **Response​​​​​​​:** *We have added a second new paragraph in the Discussion following the paragraph above, which addresses this question (among others): “There are also some challenges and open questions, for instance, whether potential safe-guards to prevent synthetic data being used in the place of real data (as discussed above). A further issue is the question of ownership; that is, who owns the synthetic data? At present, synthetic ALSPAC data is allowed to be made freely-available to all without the need to managed access or agreements to be signed. This is largely to make the synthetic data and open and accessible as possible, while minimising additional administrative work for ALSPAC staff. If needed, however, this may change in the future. An additional question is whether such synthetic datasets meet journal and funder requirements for data sharing. Although sharing synthetic data is clearly better than sharing no data, clarity from funders and journals is required to definitively answer this. However, in our experience some journals that have clear policies mandating data sharing have been open to the sharing of synthetic ALSPAC data, given that the raw ALSPAC data cannot be released.”*

 Would synthetic data meet EU GDPR data sharing guidelines, while it is artificial but may still apply (see Beduschi A et al.(2024 ^1^))   ​​​​​​​

**Response​​​​​​​:** *Based on our reading of this paper, these synthetic datasets would not need to be GDPR compliant as our synthesis methods are fully synthetic (i.e., do not retain any original individual participant data). We have now added this to the second paragraph of the Introduction: “However, as data are simulated and do not correspond to real-life individuals by design, they preserve participant anonymity (note that, because fully synthetic datasets do not contain personal information of real individuals, they are likely exempt from complying to the European Union’s General Data Protection Regulation [GDPR]; Beduschi, 2024).”*  

**Specific comments:**

- P3: When mentioning other tree-based and parametric methods beyond CART, it would help to specify some of these.  
**Response​​​​​​​:** *Now updated: “it is also possible to synthesise data using alternative tree-based (e.g., random forest) or parametric (e.g., linear, logistic) models”*
- P4. For longitudinal data how is potential disclosure managed because you now have temporal data that has the potential to identify participants?
**Response​​​​​​​:** *For longitudinal data our approach for disclosure management is still the same – i.e., remove participants if all their data are uniquely replicated in both the original and synthetic datasets. We do not believe that longitudinal/repeated data requires a different approach to disclosure compared with cross-sectional data.*
- P5. More details on the specifics of the similarity of distributions. Guidance here or recommendations would be useful.
  ​​​​​​​​​​​​​​ **Response​​​​​​​:** *This point was also raised by reviewer 1. Essentially, we do not believe that it is possible to make an objective decision regarding whether synthetic data are ‘similar enough’ to the observed data to be ‘useful’. For instance, even datasets which randomly synthesise each variable independent of all others can still be useful to explore the underlying code/analysis approach and checking for errors, even if all relations between the variables are lost (see Shepherd et al., 2017;* *https://doi.org/10.1093/aje/kwx066* *). We refer this reviewer to our response to reviewer 1 for more details.*
- Figure 4: Why are so many of the Z-values 0 for the synthetic data? Is this because they are potentially categorical?
  ​​​​​​​ **Response​​​​​​​:** *Apologies for any confusion here. This is because these results are the Z-values of the point estimate and 95% confidence interval (not Z-scores of the original variable). This is the default option for ‘synthpop’, which we have now noted more clearly in the manuscript (point 7 in the step-by-step guide): “note that, by default, ‘synthpop’ converts all coefficients to z-values so that all coefficients are on the same scale and hence easier to compare”.*
- Tables: Showing the synthetic data and the observed data for each covariate on the same row may be easier to interpret for the reader.​​​​​​​​​​​​​​
  ​​​​​​​ **Response​​​​​​​:** *This was also suggested by reviewer 1, and the tables have been updated accordingly.*

# Reviewer report: Reviewer response for version 1

**DOI:** 10.21956/wellcomeopenres.22725.r95196

**Published:** 2024-08-29

**Authors:** Neal Goldstein

**Affiliations:** 1 School of Public Health, Drexel University, Philadelphia, Pennsylvania, USA

This article describes how an R package ‘synthpop’ can be used to create a synthetic population based on the ALSPAC longitudinal study. The authors observed that the synthetic population largely recapitulated analyses conducted on the original data.

 The article is well written and deals with a timely and important topic in research: reproducibility and transparency. The R code included with the article is easy to follow and should allow other researchers to implement ‘synthpop’ on their own.

**I have a few comments for additional consideration:**

1. First, on pg5, I am dubious that a claim such as “These are synthesized ALSPAC datasets, and are not suitable for research purposes” - also echoed pg 11 “we state clearly here that these synthetic datasets should not be used in place of the actual observed data for research purposes;” - will prevent researchers from conducting original research on these synthetic data as they are more readily accessed. What other precautions can the authors recommend here? Signing an agreement before download? There is also an onus on journals or reviewers when seeing a synthetic data study to understand the intention of the research.
2. Second, I would like to have seen a brief comparison of data anonymizing approaches (e.g., to scrub the protected health information from an electronic health record export) to a wholly synthetic data approach. When and under what conditions are each appropriate?
3. Third, how does use of synthpop (or other synthetic data generators) satisfy funders’ data sharing requirements? From an NIH-centric perspective, this would be their Data Management and Sharing Policy.
4. Fourth, while there is a check for replicated unique individuals, we must also be concerned about small cell counts; even though an observation could be unique, because there are so few of them it could allow for identification of individuals. This is common in surveillance data where small cell counts are suppressed.
5. Fifth, on pg4, the claim “difference in sample size should not make much difference in practice” – is potentially misleading because this could lead to an important loss in precision in estimates.

Is the rationale for developing the new method (or application) clearly explained?

Partly

Is the description of the method technically sound?

Yes

Are the conclusions about the method and its performance adequately supported by the findings presented in the article?

Yes

If any results are presented, are all the source data underlying the results available to ensure full reproducibility?

Yes

Are sufficient details provided to allow replication of the method development and its use by others?

Yes

Reviewer Expertise:

Epidemiology

I confirm that I have read this submission and believe that I have an appropriate level of expertise to confirm that it is of an acceptable scientific standard, however I have significant reservations, as outlined above.

# Response

**Published:** 2024-12-17

**Authors:** Daniel Smith

**Affiliations:** University of Bristol, UK

*We thank the reviewer for their positive review and their constructive comments. We have responded to the reviewer’s comments in turn below, with the reviewer’s original comments in standard font and our responses in italics.*   I have a few comments for additional consideration:

 First, on pg5, I am dubious that a claim such as “These are synthesized ALSPAC datasets, and are not suitable for research purposes” - also echoed pg 11 “we state clearly here that these synthetic datasets should not be used in place of the actual observed data for research purposes;” - will prevent researchers from conducting original research on these synthetic data as they are more readily accessed. What other precautions can the authors recommend here? Signing an agreement before download? There is also an onus on journals or reviewers when seeing a synthetic data study to understand the intention of the research.

**Response:** *This is a good point. We want to make these synthetic datasets as open as possible to avoid unnecessary bureaucracy and barriers to data access, so currently recommend just making these synthetic datasets openly-available (e.g., on GitHub or OSF). We are therefore trusting researchers not to abuse this system by using these synthetic datasets for research purposes.* *Nonetheless, we have put some safe-guards in place to try and mitigate this possibility, such as: including a disclaimer in any published paper and with the synthetic data warning that data are synthetic and should not be used for research purposes; reducing the number of variables and observations in the synthetic dataset so that the synthetic dataset is very specific to the research question of interest (and hence may not be applicable to other research questions), and; including a ‘FALSE_DATA’ variable in the dataset so users cannot confuse it with actual data (points 2, 6 and 7 of the ‘ALSPAC guidelines’ section). We will also develop ALSPAC-specific guidelines in the future if these rules are found to be violated.* *The paragraph in the Discussion on this has now been updated: “To try and avoid synthetic ALSPAC data being used for research purposes – whether knowingly or otherwise – we have put a number of safe-guards in place (see points 2, 6 and 7 of the ‘ALSPAC guidelines for releasing synthetic data using ‘synthpop’’ section). Additional measures will be put in place should synthetic ALSPAC data be found to have been used for these purposes.”*  

 Second, I would like to have seen a brief comparison of data anonymizing approaches (e.g., to scrub the protected health information from an electronic health record export) to a wholly synthetic data approach. When and under what conditions are each appropriate? ​​​​​​​

**Response​​​​​​​:** *While we agree a comparison of data anonymisation approaches would be informative, this is beyond the scope of the present article, which focuses specifically on using synthpop to create synthetic ALSPAC data. At present, ALSPAC does not permit data to be made available via other approaches which aim to anonymise and de-identify participants (e.g., statistical disclosure control; Templ et al., 2015:* *https://doi.org/10.18637/jss.v067.i04* *)* *as these still largely make use of observed – rather than wholly synthetic – data, although this may change in the future. We have added a sentence to this effect in the Discussion (paragraph 4).*  

 Third, how does use of synthpop (or other synthetic data generators) satisfy funders’ data sharing requirements? From an NIH-centric perspective, this would be their Data Management and Sharing Policy.

**Response​​​​​​​:** *This is a very good question, and one we are not entirely clear on the answer to! As we note in the introduction “although data sharing is still not universal, there is a continued push from academics, journals, funders and governments towards this goal”, we are not aware of any specific policies from funders regarding the sharing of synthetic data. Most funders now do recommend that data should be openly-available (if possible), so a policy of sharing synthetic data when data could not otherwise be shared should appeal to them, but without talking to – and clarification from – funders, the actual implications of this for funders’ data sharing requirements are unclear.* *We have added a section on this in the Discussion: “An additional question is whether such synthetic datasets meet journal and funder requirements for data sharing. Although sharing synthetic data is clearly better than sharing no data, clarity from funders and journals is required to definitively answer this. However, in our experience some journals that have clear policies mandating data sharing have been open to the sharing of synthetic ALSPAC data, given that the raw ALSPAC data cannot be released.”*  

 Fourth, while there is a check for replicated unique individuals, we must also be concerned about small cell counts; even though an observation could be unique, because there are so few of them it could allow for identification of individuals. This is common in surveillance data where small cell counts are suppressed.

**Response​​​​​​​:** *We agree that this is an issue for observed data (and ALSPAC already has a policy of no potentially-identifiable cell counts of less than 5 in publications;* *https://www.bristol.ac.uk/media-library/sites/alspac/documents/alspac-publications-checklist.pdf* *), but we would argue that this is less of an issue for synthetic data. As we note in our paper: “As synthetic datasets are wholly simulated, data ought to be non-disclosive as they are no longer based on individual records. However, it is possible that a unique combination of values could be synthesised corresponding to a unique individual in the observed data, thus remaining a potential disclosure risk. Although researchers using the synthesised data will not be able to know whether such a unique observation matches that of an actual participant, there is a remote possibility that unique individuals could be identified from the synthesised data. We therefore recommend that users undertake ‘statistical disclosure control’ checks on any synthetic datasets to remove any unique observations that occur in both the observed and synthetic datasets.”* *The recommendation to remove uniquely-replicated individuals is therefore likely to be over-cautious as it would be almost-impossible to know whether the data for a uniquely-identified individual in the synthetic dataset was the same as a uniquely-identified individual in the observed dataset. This risk is therefore even more remote when considering small cell counts greater than 1 or on a smaller number of variables, hence why we have focused on uniquely-replicated individuals here.*  

 Fifth, on pg4, the claim “difference in sample size should not make much difference in practice” – is potentially misleading because this could lead to an important loss in precision in estimates.

 ​​​​​​​​​​​​​​ **Response​​​​​​​:** *Yes, this is technically true – Although in practice the differences in sample sizes between observed and synthetic data are generally small, so should make little difference in reality. Nonetheless, we have updated this sentence accordingly: “this difference in sample size should not make much difference in practice (although large differences in sample size may lead to a loss of precision in estimates)”.*

# Reviewer report: Reviewer response for version 1

**DOI:** 10.21956/wellcomeopenres.22725.r79973

**Published:** 2024-05-17

**Authors:** Sam Harper

**Affiliations:** 1 Department of Epidemiology, Biostatistics and Occupational Health, School of Population and Global Health, McGill University, Montreal, Canada

-  General Comments

- This paper provides a sensible overview and introduction to the practical use of synthetic datasets in the service of increasing the transparency and reproducibility of epidemiologic research. Overall I think the paper is generally well written and structured, but I also have a few comments and suggestions. 

 - As a general comment, the authors (in many places) make it clear that deciding 'how good is good enough' for synthetic data requires subjective interpretations. However, I think readers would probably benefit from more discussion of how to describe, evaluate, or measure the concept of 'faithfulness ' between the synthetic and observed datasets and statistical relationships. Are there no rules of thumb or guidance about how to compare, say, measures of central tendency or variability in ways that would help researchers to draw sensible conclusions about comparability? If not, perhaps some could or should even be suggested here? 

- Specific comments

- p4. The authors note that the order in which variable are synthesized may also affect 'correspondence' between synthesized and observed data. Is this a potential consequence of not respecting the temporal ordering of the variables in question? And is this more important for causal questions than, say, descriptive or predictive analyses? For example, if both income and education are included, wouldn't it be prudent to synthesize education first, since that is a potential determinant of income? Should researchers use a directed acyclic graph in order to make these relationships clear (if the analysis is causal)?

 - p4. The authors say that synthesizing the 'exposures and outcomes last' is advantageous, but at this point in the paper it isn't clear what the exposure or outcome is, since there has only been talk about ALSPAC more generally. This makes me wonder whether the authors would provide different guidance for those wanting to produce a synthetic dataset for a specific exposure-outcome investigation vs. a synthetic general dataset for multiple uses. Would this lead to different strategies for implementation?

 - p4. "We advise that users test different synthesizing methods, and see what works best for their specific dataset", but how is 'works best' defined here? By some metric?

 - p5. "Check that the distributions are similar..." how similar (e.g., a standardized difference of <10%? Means or medians within a certain range?). Again, a little help or guidance here could be beneficial, even if no hard-and-fast rules apply. I think for many readers that have never heard of the concept of generating synthetic data, this would help.

 - p7 (Figure 4). I recognize that the purpose of this figure is to demonstrate the similarity between the coefficients in the synthetic and observed data (which it does), but it's important to make it clear to readers that in an analysis with a focal exposure of interest and and outcome, these coefficients do not have any straightforward interpretation (i.e., Table 2 fallacy from Westreich et al 2013 AJE), and any direct interpretations should be avoided. 

 - p9. I know that it is impossible to put all of the details for both cross-sectional and longitudinal synthetic datasets together, but I think at least some intuition for how the process of developing the synthetic data for the longitudinal case would be of interest. Was this done parametrically or again by regression trees? How to deal with constraints placed on some of the longitudinal variables (e.g., some can only increase, like age or education). A few details would probably be helpful. 

 - p11. "the main effect of sex did vary between the observed and synthetic data" By how much? Meaningfully? Also a little confusing because in the context of EMM there is no 'single' effect of sex (i.e., effect of sex at what age?) so it isn't clear what "main effect of sex" means here.

 - p11. Also the authors suggest this could be due to the removal of 200 individuals due to SDC, but can they possibly confirm that this is the case (even if, ultimately those individuals would have to be removed in order to make the synthetic dataset available?). At least you could rule this out as an explanation. 

 - p14. I'm a little confused regarding the comment on "multi-level/ hierarchical data" as a limitation.  ALSPAC is multi-level data, right (occasions nested within individuals)? So were the observations just treated as independent for the longitudinal / trajectory models given above? Does that have any consequences for variance estimation using the synthetic vs. the observed data? 

-  Minor comments

- Figure 2 is great but hard to read. Would be better to increase the font size for the legend as well as for each strip text heading and x- and y-axes (including titles). Could also drop the grayscale background but that is more of a personal preference.

 - Figure 3. Not quite sure that it is best to use the Z-value for the x-axis. Might someone confuse this with a z-statistic (i.e., from a test?). Why not just use the regression coefficient, or clarify that this is a symptom score?

 - Table 1. I would encourage re-orienting the table so that readers can look to compare coefficients and standard errors from left to right to compare synthetic vs. observed values across columns, rather than across rows. It would be much easier to read. 

 - Table 4. Would also encourage re-orienting to compare synthetic vs. observed across columns rather than rows.

Is the rationale for developing the new method (or application) clearly explained?

Yes

Is the description of the method technically sound?

Partly

Are the conclusions about the method and its performance adequately supported by the findings presented in the article?

Yes

If any results are presented, are all the source data underlying the results available to ensure full reproducibility?

Yes

Are sufficient details provided to allow replication of the method development and its use by others?

Yes

Reviewer Expertise:

epidemiology, impact evaluation, reproducible research

I confirm that I have read this submission and believe that I have an appropriate level of expertise to confirm that it is of an acceptable scientific standard, however I have significant reservations, as outlined above.

# Response

**Published:** 2024-12-17

**Authors:** Daniel Smith

**Affiliations:** University of Bristol, UK

*We thank the reviewer for their positive review and their constructive comments. We also apologise for the length of time taken to respond to the reviewer’s comments; it took quite a while to find additional reviewers for our paper, and we thank this reviewer for their patience. We have responded to the reviewer’s comments in turn below, with the reviewer’s original comments in standard font and our responses in italics.*  

 As a general comment, the authors (in many places) make it clear that deciding 'how good is good enough' for synthetic data requires subjective interpretations. However, I think readers would probably benefit from more discussion of how to describe, evaluate, or measure the concept of 'faithfulness ' between the synthetic and observed datasets and statistical relationships. Are there no rules of thumb or guidance about how to compare, say, measures of central tendency or variability in ways that would help researchers to draw sensible conclusions about comparability? If not, perhaps some could or should even be suggested here?

**Response:** *While we sympathise with the reviewer’s sentiments here, we do not believe that it is possible to make an objective decision regarding whether synthetic data are ‘similar enough’ to the observed data to be ‘useful’. For instance, even datasets which randomly synthesise each variable independent of all others can still be useful to explore the underlying code/analysis approach and checking for errors, even if all relations between the variables are lost (see Shepherd et al., 2017;* *https://doi.org/10.1093/aje/kwx066* *). Of course, the closer the synthetic data are to the observed the better, but in the absence of clear dividing lines, and different needs depending on the type of dataset, we do not feel it would be appropriate for us to provide guidance on this to readers.* *In our original manuscript we pointed readers to the concept of ‘utility’, which measures how similar the synthetic and observed data are, but did not develop this further for the reasons outlined above (see paragraph 4 of the ‘* *Recommendations when using ‘synthpop’ section): “The second factor to consider is whether the synthetic dataset successfully maintains relations between variables (although even synthetic datasets which do not maintain relations between variables can still be useful for reproducing code and checking for errors; (Shepherd et al., 2017)). The ‘synthpop’ package provides a suite of useful tools for comparing the synthesised data against the observed data. This includes a simple comparison of the distributions of each variable, through to more complex conditional associations, such as in a multivariable regression. Ideally, these should be similar between the observed and synthetic datasets, although random variation and imperfections in the synthesising process are of course inevitable. Formal measures of ‘utility’, comparing the synthetic to the observed data, are also available within the ‘synthpop’ package, but are not discussed here (Raab et al., 2017). […] There are no definitive rules on what constitutes ‘successful’ synthesis, so again researchers must use their subjective judgement.”*  

**Specific comments** P4. The authors note that the order in which variable are synthesized may also affect 'correspondence' between synthesized and observed data. Is this a potential consequence of not respecting the temporal ordering of the variables in question? And is this more important for causal questions than, say, descriptive or predictive analyses? For example, if both income and education are included, wouldn't it be prudent to synthesize education first, since that is a potential determinant of income? Should researchers use a directed acyclic graph in order to make these relationships clear (if the analysis is causal)? **Response:** *As synthpop models are predictive, rather than based on the causal structure/data-generating mechanisms, it does not appear necessary to take the (assumed) causal relations between variables into consideration when synthesising data (e.g., in the reviewer’s example, whether education is synthesised before income, or income before education, would make little difference to the final synthetic dataset).* *We have added the following sentence to see paragraph 5 of the ‘* *Recommendations when using ‘synthpop’ section to clarify this: “Note that as synthetic data generation using ‘synthpop’ uses predictive modelling, it is not necessary to consider the causal structure of the data (i.e., the data-generating mechanisms) when synthesising data using this approach.”*

 p4. The authors say that synthesizing the 'exposures and outcomes last' is advantageous, but at this point in the paper it isn't clear what the exposure or outcome is, since there has only been talk about ALSPAC more generally. This makes me wonder whether the authors would provide different guidance for those wanting to produce a synthetic dataset for a specific exposure-outcome investigation vs. a synthetic general dataset for multiple uses. Would this lead to different strategies for implementation?

**Response:** *We have added further clarification on this in brackets following this sentence: “While synthesising the data in any order may be sufficient, we have found that synthesising the exposure(s) and outcome(s) last sometimes maintains the relations between variables more faithfully, although this does somewhat contradict the advice given in (Raab et al., 2017), who recommend synthesising the most important variables first (although if the dataset is being synthesised for more general use and does not have specific exposures or outcomes, synthesising the variables in any order will likely suffice).”*

 p4. "We advise that users test different synthesizing methods, and see what works best for their specific dataset", but how is 'works best' defined here? By some metric? **Response:** *Again, we appreciate that these decisions are somewhat vague and ‘vibes-y’, but this is inevitable when dealing with subjective judgements such as this. However, we have now clarified this statement and provided an example to help readers: “We advise that users test different synthesising methods, and see what works best for their specific dataset in terms of replicating the properties of the observed data (e.g., one method may maintain the relationship between the exposure and outcome more faithfully than another).”*

 p5. "Check that the distributions are similar..." how similar (e.g., a standardized difference of <10%? Means or medians within a certain range?). Again, a little help or guidance here could be beneficial, even if no hard-and-fast rules apply. I think for many readers that have never heard of the concept of generating synthetic data, this would help.

**Response:** *While we do not propose any quantitative measures to assess similarity for the reasons outlined above, we have added that simply eye-balling the data is generally sufficient for this: “Check that the distributions of all synthesised variables are similar to those in the observed data. Simply visualising the data (e.g., in bar charts or histograms) should provide this information (see worked example below).”*

 p7 (Figure 4). I recognize that the purpose of this figure is to demonstrate the similarity between the coefficients in the synthetic and observed data (which it does), but it's important to make it clear to readers that in an analysis with a focal exposure of interest and and outcome, these coefficients do not have any straightforward interpretation (i.e., Table 2 fallacy from Westreich et al 2013 AJE), and any direct interpretations should be avoided. **Response:** *We have added the following text to make this clear to readers: “we also stress here that in a causal analysis between the exposure and outcome the coefficients of these additional covariates do not have a straightforward interpretation [see Westreich and Greenland, 2013 on the ‘table 2’ fallacy]).”*

 p9. I know that it is impossible to put all of the details for both cross-sectional and longitudinal synthetic datasets together, but I think at least some intuition for how the process of developing the synthetic data for the longitudinal case would be of interest. Was this done parametrically or again by regression trees? How to deal with constraints placed on some of the longitudinal variables (e.g., some can only increase, like age or education). A few details would probably be helpful. 

**Response:** *We have provided additional details of the synthesis approach used in the ‘Applied longitudinal example’ section: “For both examples, we synthesised datasets using the default CART approach with these longitudinal data in ‘wide’ format (i.e., one row per participant with time-points as separate variables); synthesised data were then converted to ‘long’ format for the multilevel modelling analysis (i.e., multiple observations per participant with one row per time-point).”* *Additional details of the data used for the repeated measures analyses have also been provided (new text in bold font): “*The first example examines height trajectories from childhood to early adulthood using a multilevel modelling framework, similar to that of Howe *et al.* ( Howe *et al.*, 2012; Howe *et al.*, 2016), **using up to eight occasions of height between approximately 7 to 18 years of age**. The second example uses growth mixture modelling to examine associations between adolescent self-esteem and depression trajectories across adolescence and early adulthood, and then depression trajectories across adolescence and early adulthood associated with later depression, similar to that of Kwong *et al.* ( Kwong *et al.*, 2019) and López-López *et al.* ( López-López *et al.*, 2020), **using up to nine occasions of depressive symptoms between approximately 10 and 24 years of age**. *”* *We have also updated the discussion to be clearer on when the standard ‘synthpop’ package can be used for multi-level/hierarchical data and when it cannot: “For instance, when using the standard ‘synthpop’ package, synthesising data in ‘wide’ format (as used here) appears to work well, but is unlikely to hold for data in ‘long’ format as information on the relations between observations within individuals would be lost.”*

 p11. "the main effect of sex did vary between the observed and synthetic data" By how much? Meaningfully? Also a little confusing because in the context of EMM there is no 'single' effect of sex (i.e., effect of sex at what age?) so it isn't clear what "main effect of sex" means here. **Response:** *We have now clarified this in the text: “the main effect of sex (i.e., the difference in height between males and females at age 12 [the mean age of all the assessments]) did vary between the observed and synthetic data”. This information has also been added to the footnotes in Tables 1 and 2.* *As we note later in this paragraph, this did not seem to meaningfully impact on differences between the synthetic and observed data: “However, as shown in Figure 6, this had little effect on the estimation of the height trajectories and it is worth noting the confidence intervals for the main effect overlap”.*

 p11. Also the authors suggest this could be due to the removal of 200 individuals due to SDC, but can they possibly confirm that this is the case (even if, ultimately those individuals would have to be removed in order to make the synthetic dataset available?). At least you could rule this out as an explanation. 

**Response:** *We have re-run the analysis without including the SDC command in ‘synthpop’ and find that the results are nearly identical to the analysis that contain the SDC command in ‘synthpop’, suggesting that it is not the removal of the SDC observations, but probably more likely random variability from the synthesis model. These results presented in the table below.* *We have now amended the manuscript to reflect this: “However, the main effect of sex (i.e., the difference in height between males and females at age 12 [the mean age of all the assessments]) did vary between the observed and synthetic data, which is likely a result of random variability in the synthesis model”.*

     Synthetic with SDC command ( *n*=9,834, *n _obs_* =52,557) Synthetic without SDC command ( *n*=9,969, *n _obs_* =52,692) **Fixed effects** *Beta* *Std Err* *95% CI* *P* *Beta* *Std Err* *95% CI* *P*    Age 5.65 0.01 5.63 to 5.67 <0.001 5.65 0.01 5.63 to 5.67 <0.001    Female -0.35 0.14 -0.63 to -0.06 0.016 -0.35 0.13 -0.63 to -0.06 0.014    Female*Age -0.99 0.01 -1.02 to -0.97 <0.001 -0.99 0.01 -1.02 to -0.96 <0.001    Age ^2^ -0.12 0.00 -0.12 to -0.11 <0.001 -0.12 0.00 -0.12 to -0.11 <0.001    Female*Age ^2^ -0.26 0.00 -0.27 to -0.25 <0.001 -0.26 0.00 -0.27 to -0.25 <0.001    Intercept 153.00 0.10 152.80 to 153.19 <0.001 153.00 0.10 152.80 to 153.19 <0.001 **Random effects** *Estimate* *Std Err* *95% CI* *Estimate* *Std Err* *95% CI*      var(Age) 0.17 0.01 0.16 to 0.18   0.17 0.01 0.16 to 0.18      var(Age ^2^) 0.01 0.00 0.01 to 0.01   0.01 0.00 0.01 to 0.01      var(Intercept) 45.20 0.71 43.83 to 46.62   45.20 0.71 43.83 to 46.62      cov(Age, Age ^2^) 0.02 0.00 0.01 to 0.02   0.02 0.00 0.01 to 0.02      cov(Age, Intercept) 0.77 0.05 0.68 to 0.87   0.77 0.05 0.68 to 0.87      cov(Age ^2^, Intercept) -0.53 0.01 -0.55 to -0.50   -0.53 0.01 -0.55 to -0.50      var(Residual) 7.54 0.06 7.42 to 7.67   7.54 0.06 7.42 to 7.67    

 p14. I'm a little confused regarding the comment on "multi-level/ hierarchical data" as a limitation.  ALSPAC is multi-level data, right (occasions nested within individuals)? So were the observations just treated as independent for the longitudinal / trajectory models given above? Does that have any consequences for variance estimation using the synthetic vs. the observed data? *We have hopefully explained this above, re. discussion of ‘wide’ vs ‘long’ longitudinal data formats.* *In terms of variance estimation, there does not appear to be any large, consistent or meaningful differences between the variances, standard errors and random effects for the observed vs synthetic data (Tables 1-4), suggesting that this is not an issue (at least when synthesising longitudinal/repeated measures data in ‘wide’ format).* **Minor comments**

 Figure 2 is great but hard to read. Would be better to increase the font size for the legend as well as for each strip text heading and x- and y-axes (including titles). Could also drop the grayscale background but that is more of a personal preference.

**Response:** *These plots are the default ones created by the ‘synthpop’ package, and are surprisingly difficult to edit manually! To try and make this figure easier to read, we have changed the orientation from landscape (3 rows, 5 columns) to portrait (5 rows, 3 columns) which should increase the plot size in the manuscript and make these easier to read.*

 Figure 3. Not quite sure that it is best to use the Z-value for the x-axis. Might someone confuse this with a z-statistic (i.e., from a test?). Why not just use the regression coefficient, or clarify that this is a symptom score?

**Response:** *This is the default option for ‘synthpop’, which we have now noted more clearly in the manuscript (point 7 in the step-by-step guide): “note that, by default, ‘synthpop’ converts all coefficients to z-values so that all coefficients are on the same scale and hence easier to compare”.*

 Table 1. I would encourage re-orienting the table so that readers can look to compare coefficients and standard errors from left to right to compare synthetic vs. observed values across columns, rather than across rows. It would be much easier to read. 

 Table 4. Would also encourage re-orienting to compare synthetic vs. observed across columns rather than rows.

**Response:** *We have re-formatted Tables 1, 2 and 4 to make the comparison between synthetic and observed results easier.*
